# Prevalence of Oral Human Papillomavirus Infection Among Urban Gay, Bisexual, and Other Men Who Have Sex With Men in Canada, 2017–2019

**DOI:** 10.1093/infdis/jiae345

**Published:** 2024-07-23

**Authors:** Jenna Alessandrini, Joseph Cox, Alexandra de Pokomandy, Trevor A Hart, Daniel Grace, Troy Grennan, David Moore, Gilles Lambert, Catharine Chambers, Shelley L Deeks, Ramandip Grewal, Nathan J Lachowsky, Chantal Sauvageau, Darrell H S Tan, François Coutlée, Ann N Burchell, the Engage-HPV Team, Jody Jollimore, Rosane Nisenbaum, Gina Ogilvie, Daniel Grace, Trevor Hart, Joseph Cox, Gilles Lambert, Jody Jollimore, Nathan Lachowsky, David Moore, Ann Burchell, Troy Grennan, Alexandra de Pokomandy

**Affiliations:** Dalla Lana School of Public Health, University of Toronto, Toronto, Ontario, Canada; Department of Epidemiology, Biostatistics, and Occupational Health, McGill University, Montréal, Quebec, Canada; Research Institute of the McGill University Health Centre, Montréal, Quebec, Canada; Direction de Santé Publique de Montréal, Montréal, Quebec, Canada; Research Institute of the McGill University Health Centre, Montréal, Quebec, Canada; Department of Family Medicine, McGill University, Montréal, Quebec, Canada; Dalla Lana School of Public Health, University of Toronto, Toronto, Ontario, Canada; Department of Psychology, Toronto Metropolitan University, Toronto, Ontario, Canada; Dalla Lana School of Public Health, University of Toronto, Toronto, Ontario, Canada; British Columbia Centre for Disease Control, Vancouver, British Columbia, Canada; British Columbia Centre for Excellence in HIV/AIDS, Vancouver, British Columbia, Canada; Direction de Santé Publique de Montréal, Montréal, Quebec, Canada; Dalla Lana School of Public Health, University of Toronto, Toronto, Ontario, Canada; MAP Centre for Urban Health Solutions, Unity Health Toronto, Toronto, Ontario, Canada; Department of Health and Wellness, Nova Scotia, Halifax, Nova Scotia, Canada; Department of Community Health and Epidemiology, Dalhousie University, Halifax, Nova Scotia, Canada; Dalla Lana School of Public Health, University of Toronto, Toronto, Ontario, Canada; School of Public Health and Social Policy, University of Victoria, Victoria, British Columbia, Canada; Immunization, Institut National de Santé Publique du Québec, Quebec, Quebec, Canada; CHU de Québec-Université Laval Research Center, Quebec, Quebec, Canada; MAP Centre for Urban Health Solutions, Unity Health Toronto, Toronto, Ontario, Canada; Department of Epidemiology, Biostatistics, and Occupational Health, McGill University, Montréal, Quebec, Canada; Départements de Médecine et de Médecine de Laboratoire, Service de Microbiologie et Infectiologie, Centre Hospitalier de l’Université de Montréal, Montréal, Quebec, Canada; Département de Microbiologie, Infectiologie et Immunologie, Université de Montréal, Montréal, Quebec, Canada; MAP Centre for Urban Health Solutions, Unity Health Toronto, Toronto, Ontario, Canada; Department of Family and Community Medicine, Unity Health Toronto, Toronto, Ontario, Canada

**Keywords:** oral human papillomavirus, gay, bisexual, and other men who have sex with men, vaccine effectiveness

## Abstract

**Background:**

Oral human papillomavirus (HPV) infections are a leading cause of oropharyngeal cancers. In 2015 and 2016, HPV vaccines became publicly funded for gay, bisexual, and other men who have sex with men (GBM) under 27 years of age in most Canadian provinces.

**Methods:**

Between 2017 and 2019, sexually active GBM in Montreal, Toronto, and Vancouver were recruited through respondent-driven sampling. Participants aged 16–30 years were invited to self-collect oral rinse specimens for HPV testing. We estimated HPV prevalence in the oral tract overall and compared these by vaccination status.

**Results:**

Among the 838 GBM with a valid oral specimen, 36.9% reported receiving ≥1 dose of HPV vaccine. Overall, oral HPV prevalence was 2.6% (95% confidence interval [CI], 1.5%–3.7%) for at least 1 HPV type and 1.2% (95% CI, .5%–1.9%) for any high-risk type. We detected quadrivalent (HPV 6/11/16/18) vaccine-preventable types in 0.3% (95% CI, .0%–1.0%) of vaccinated individuals and 1.1% (95% CI, .1%–2.0%) of unvaccinated individuals.

**Conclusions:**

Oral HPV prevalence was low in a population of young urban GBM in Canada, of whom 37% were vaccinated. Findings serve as a benchmark for monitoring of vaccination impacts on oral HPV infection within this priority population.

## Teaser

In a cohort of young Canadian gay, bisexual, and other men who have sex with men (approximately 37% vaccinated), prevalence of oral human papillomavirus infection was relatively low overall with estimates higher for those unvaccinated compared to those vaccinated.

Human papillomavirus (HPV) is one of the most common sexually transmitted infections (STIs) worldwide [1]. In addition to its causal role in anogenital cancers, high-risk oncogenic HPV types (HPV 16/18/31/33/35/39/45/51/52/56/58/59/68) are a cause of head and neck cancers, specifically oropharyngeal squamous cell carcinomas (OPCs) [2]. Attributable fractions for high-risk HPV and OPCs are estimated to be 31% globally [3] and greater in high-income countries such as Canada where HPV is responsible for over 70% of OPCs [4]. Cancer surveillance has revealed an increasing incidence of OPCs over time, largely among younger males (<60 years) [5]. Evidence regarding vaccine effectiveness (VE) for oral HPV infection [6–9] and associated disease is limited. However, VE has previously been reported as high against persistent HPV-type infections, and precancerous and cancer outcomes in anogenital sites [10, 11]. This is particularly the case for those vaccinated at a younger age [12], indicating the importance of vaccination earlier on in life, primarily before sexual exposure.

Gay, bisexual, and other men who have sex with men (GBM) are considered a priority population for HPV vaccination [13] given their high burden of HPV-related disease, notably anal cancer [14, 15]. In a meta-analysis conducted using data from largely unvaccinated populations worldwide between 2007 and 2013, the pooled prevalence of oral high-risk HPV infections was 9.1% (95% confidence interval [CI], 4.0%–14.2%) among GBM without human immunodeficiency virus (HIV) and 16.5% (95% CI, 8.2%–24.8%) among GBM with HIV, with overall prevalence increasing with age [16].

In 2015 and 2016, targeted programs offering publicly funded HPV vaccination for GBM 9–26 years of age were initiated in Canada's 3 most populous provinces: British Columbia [17], Ontario [18], and Québec [19]. Two vaccines have been authorized for use in males 9–26 years of age in Canada: Gardasil, used for the prevention of HPV types 6/11/16/18, and Gardasil 9, used for prevention of HPV types 6/11/16/18/31/33/45/52/58 [20] (Cervarix has also been administered as part of school-based and catch-up programs in Québec [21] and British Columbia [22] respectively; however, not for the targeted GBM program). The nonavalent (9vHPV) vaccine replaced the quadrivalent (4vHPV) vaccine with gradual roll-out starting in January 2016 in Québec, May 2017 in British Columbia, and September 2017 in Ontario.

To evaluate the impacts of such programs and guide future vaccine uptake, particularly among younger GBM who are at a substantially higher risk for HPV-associated OPCs, it is important to monitor the burden of HPV infections in these priority populations. Here we report findings from a sample of young GBM aged 16–30 years living in Vancouver, British Columbia; Toronto, Ontario; and Montreal, Québec, to estimate the prevalence of oral HPV infections, with comparisons by vaccination status, city, and age.

## METHODS

### Study Design, Participants, Recruitment, and Data Collection

We carried out a cross-sectional analysis of baseline data from participants of Engage, a community-based prospective sexual cohort health study of GBM in Montreal, Vancouver, and Toronto, Canada. The study links epidemiological and behavioral data with laboratory-based indicators of various STIs. A total of 2449 self-identified GBM (cisgender and transgender) aged ≥16 years, who reported having sex with another man within the past 6 months, read English or French, and consented to participate, were recruited through respondent-driven sampling (RDS) [23] between February 2017 and August 2019. Briefly, RDS involves nonrandomly selecting initial participants (“seeds”) to represent various traits/subgroups of the GBM community; seeds were asked to recruit up to 6 members of their social networks, who if eligible and participated, were then asked to recruit members of their social networks. This incentivized recruitment continued through waves until the target sample size was reached in each city. Recruitment has been described in detail elsewhere [24, 25]. We used the STROBE-RDS checklist to guide reporting [26]. Using computer-assisted self-interview, participants completed a questionnaire as part of Engage; it included HPV-specific questions such as knowledge of the vaccine and vaccination status including number of doses and age at first dose. Participants received compensation for their visit (CA$60) and for each participant recruited (CA$15).

As part of the Engage-HPV substudy, individuals aged 16–30 years were invited to provide oral rinse specimens for HPV testing at enrolment starting in March, July, and August 2017, in Montreal, Vancouver, and Toronto, respectively. The study received ethical approval from Toronto Metropolitan University, University of Toronto, St Michael's Hospital, University of Windsor, University of British Columbia, University of Victoria, Simon Fraser University, and the Research Institute-McGill University Health Centre.

### Oral Human Papillomavirus Status

Men self-collected oral specimens at study sites using a Scope mouthwash rinse that has previously been validated to provide a high yield of DNA [27]. Briefly, participants were instructed to vigorously rinse the oral cavity with the mouthwash then perform gargles for 4–5 seconds to rinse the throat. DNA was extracted from the specimen as described previously [28]. We used an in-house generic probe assay to screen oral rinse specimens for the presence of HPV [29]. Positive specimens were then genotyped for 36 HPV genotypes through polymerase chain reaction-based Linear Array assay (Roche Molecular Systems) [30]. Specimen adequacy was determined by performing coamplification of a β-globin human DNA sequence. Detection of either HPV DNA or β-globin indicated that an oral specimen was valid.

### Exposures of Interest

For HPV vaccination status, our primary exposure of interest, we conservatively assumed that most vaccinated participants at enrolment received the quadrivalent Gardasil because enrolment into this study began in mid-2017, during the same timeframe when the 9vHPV vaccine replaced the 4vHPV vaccine, as described above (vaccine brand data not captured in questionnaire). Although gender-neutral school-based HPV vaccination programs in Ontario, Québec, and British Columbia were introduced in 2016/2017, the participants enrolled in our study would not have been eligible for such programs given that they are outside these birth cohorts. We compared participants with a self-reported receipt of ≥1 dose of the HPV vaccine with those with a self-reported receipt of 0 doses. We assumed that participants unaware of the HPV vaccine were unvaccinated, as we have done previously [31, 32]. Other covariates of interest included age and city.

### Analyses

We restricted our analysis to participants with valid oral specimens as described above. We calculated individual HPV type-specific prevalence as well as grouped oral prevalence as defined by any HPV type, high-risk oncogenic types, low-risk types (includes non–high-risk types), 9vHPV-preventable types, and 4vHPV-preventable types. Analyses were conducted overall and stratified by city, vaccination status, and age at enrolment.

We described baseline characteristics and compared these by vaccination status. Categorical variables were summarized using counts and proportions and continuous variables with medians and interquartile ranges. Prevalence estimates with 95% Wald CIs for each outcome were calculated through dividing the number of infections by the total number of participants with a valid sample. Jeffreys Bayesian CIs were used for numerators of zero [33]. Due to small cell (single digit) counts for numerators, we reported oral HPV prevalence estimates unweighted for the RDS design because application of RDS-II weights [34] produced highly imprecise estimates. To estimate significance of all associations, 2-sided *P* values were obtained using χ^2^ tests, Fisher exact tests, Wilcoxon-rank sum tests, or Kruskal-Wallis tests where appropriate. Unknown and missing values were excluded from *P* value calculations. Finally, we carried out unadjusted logistic regression analyses between vaccination status and oral HPV infection with vaccine-preventable types to estimate odds ratios (OR) and 95% CIs; we were unable to conduct multivariable models due to the rarity of oral HPV infection. We derived VE against prevalent oral infection with vaccine-preventable HPV types as (1 − unadjusted OR) × 100%. All statistical analyses were performed in SAS and a *P* value of α < .05 was considered statistically significant.

## RESULTS

Of the 2449 GBM enrolled in Engage, there were 1003 individuals aged 16–30 years, of whom 845 (84.2%) provided an oral specimen, of whom 99.2% had a valid sample (n = 838) (Supplementary Figure 1). Individuals who provided an oral specimen were similar to those who did not provide a specimen with respect to HPV vaccination status, sexual behaviors, and most sociodemographic and health care characteristics, with the exception of city and ethnicity (Supplementary Table 1).

Of the 838 GBM included in the analysis, 46.4% were from Montreal (n = 389), 25.3% from Toronto (n = 212), and 28.3% from Vancouver (n = 237) (Table 1). Among all cities combined, the median age was 26 years (interquartile range [IQR], 23–28 years; Table 1). The majority self-identified as gay (78.6%), few were people with HIV (5.4%), almost half were current smokers (46.6%), and 35.0% ever had vaginal, oral, or anal sex with a woman. Regarding oral sex behaviors, the median age at first oral sex with a man was 17 years (IQR, 15–19 years). In the past 6 months, 96.9% and 65.9% performed oral sex and rimming on a man, respectively. Most had between 2 and 5 oral or anal male partners in the past 6 months (37.4%), followed by >10 partners (26.5%), 6–10 partners (22.1%), and 0–1 partners (14.1%). (Note that the Engage questionnaire did not capture kissing behaviors.)

**Table 1.** Characteristics of Gay, Bisexual, and Other Men Who Have Sex With Men Aged 16–30 Years at Enrolment, Overall and by HPV Vaccination Status, Engage, Canada, 2017 to 2019

Characteristic	Overall^a^ (n = 838)	Unvaccinated^b^ (n = 472)	Vaccinated^c^ (n = 309)	*P* ^d^
Age, y				**<.0001**
16–26	459 (54.8)	225 (47.7)	201 (65.0)	
27–30	379 (45.2)	247 (52.3)	108 (35.0)	
Median (IQR)	26 (23–28)	27 (24–29)	25 (23–27)	**<.0001^e^**
City				**.040**
Montreal	389 (46.4)	230 (48.7)	126 (40.8)	
Toronto	212 (25.3)	121 (25.6)	80 (25.9)	
Vancouver	237 (28.3)	121 (25.6)	103 (33.3)	
Highest level of education received				.325
High school or less	153 (18.3)	92 (19.5)	49 (15.9)	
Postsecondary	545 (65.0)	296 (62.7)	209 (67.6)	
Graduate or professional degree	140 (16.7)	84 (17.8)	51 (16.5)	
Annual pretax income, all sources, Canadian dollar				.994
<$20 000	358 (42.7)	199 (42.2)	131 (42.4)	
$20 000–$39 999	265 (31.6)	151 (32.0)	97 (31.4)	
$40 000–$59 999	143 (17.1)	81 (17.2)	56 (18.1)	
$60 000–$79 999	55 (6.6)	32 (6.8)	19 (6.1)	
$80 000+	17 (2.0)	9 (1.9)	6 (1.9)	
Ethnicity				**.019**
Indigenous	13 (1.6)	10 (2.1)	3 (1.0)	
Black, African, Caribbean	22 (2.6)	15 (3.2)	7 (2.3)	
Asian	90 (10.7)	53 (11.2)	30 (9.7)	
English or French Canadian	393 (46.9)	195 (41.3)	165 (53.4)	
Other European	154 (18.4)	93 (19.7)	57 (18.4)	
Other or mixed	166 (19.8)	106 (22.5)	47 (15.2)	
Sexual orientation				.435
Gay	659 (78.6)	371 (78.6)	250 (80.9)	
Bisexual, queer, straight, questioning, asexual, pansexual, 2-spirit	179 (21.4)	101 (21.4)	59 (19.1)	
Gender identity				.755
Cisgender man	766 (91.4)	436 (92.4)	283 (91.6)	
Transgender man	15 (1.8)	6 (1.3)	6 (1.9)	
Gender queer, gender nonconforming, 2-spirit	57 (6.8)	30 (6.4)	20 (6.5)	
Has a primary health care provider				**<.0001**
Yes	501 (59.8)	258 (54.7)	213 (68.9)	
No	337 (40.2)	214 (45.3)	96 (31.1)	
Current smoking status				**.002**
Never smoker	256 (30.8)	125 (26.7)	115 (37.6)	
Current smoker	388 (46.6)	238 (50.7)	121 (39.5)	
Former smoker	188 (22.6)	106 (22.6)	70 (22.9)	
Alcohol risk, ASSIST score^f^				**.008**
Lower risk	522 (64.7)	298 (65.6)	192 (64.4)	
Moderate risk	240 (29.7)	122 (26.9)	98 (32.9)	
High risk	45 (5.6)	34 (7.5)	8 (2.7)	
Cannabis use, lifetime				.409
Yes	711 (85.6)	393 (84.2)	265 (86.3)	
No	120 (14.4)	74 (15.8)	42 (13.7)	
Poppers use, lifetime				**.001**
Yes	458 (55.2)	238 (51.1)	193 (62.9)	
No	371 (44.8)	228 (48.9)	114 (37.1)	
Tested for STIs, lifetime				**<.0001**
Yes	751 (90.5)	398 (85.6)	299 (96.8)	
No	79 (9.5)	67 (14.4)	10 (3.2)	
Self-reported STI diagnosis, lifetime^g^				**<.0001**
Yes	453 (55.2)	217 (47.5)	214 (69.7)	
No	367 (44.8)	240 (52.5)	93 (30.3)	
Laboratory-confirmed HIV infection				.691
Positive	45 (5.4)	23 (4.9)	17 (5.5)	
Negative	789 (94.6)	447 (95.1)	290 (94.5)	
Ever had oral sex with a man, giving or receiving, lifetime				1.000^h^
Yes	837 (99.9)	471 (99.8)	309 (100.0)	
No	1 (0.1)	1 (0.2)	0 (0.0)	
Age at first oral sex with a man, giving or receiving, y				.576
Younger than 17	390 (46.7)	214 (45.5)	147 (47.6)	
17–30	446 (53.3)	256 (54.5)	162 (52.4)	
Median age (IQR)	17 (15–19)	17 (15–19)	17 (15–19)	.755^e^
Ever had sex with a woman, lifetime^i^				**.041**
Yes	293 (35.0)	174 (36.9)	92 (29.8)	
No	545 (65.0)	298 (63.1)	217 (70.2)	
No. of male sex partners, past 6 mo^j^				**.003**
0–1	104 (12.4)	64 (13.6)	30 (9.7)	
2–5	301 (35.9)	184 (39.0)	92 (29.8)	
6–10	189 (22.6)	104 (22.0)	76 (24.6)	
>10	244 (29.1)	120 (25.4)	111 (35.9)	
No. of male oral/anal sex partners, past 6 mo				**.001**
0–1	118 (14.1)	72 (15.3)	35 (11.3)	
2–5	313 (37.4)	192 (40.7)	97 (31.4)	
6–10	185 (22.1)	105 (22.2)	72 (23.3)	
>10	222 (26.5)	103 (21.8)	105 (34.0)	
Current regular partner				.812
Yes	389 (46.4)	221 (46.8)	142 (46.0)	
No	449 (53.6)	251 (53.2)	167 (54.0)	
Oral sex given to a man, past 6 mo				.138
Yes	812 (96.9)	454 (96.2)	303 (98.1)	
No	26 (3.1)	18 (3.8)	6 (1.9)	
Rimming given to a man, past 6 mo				**.001**
Yes	552 (65.9)	295 (62.5)	228 (73.8)	
No	286 (34.1)	177 (37.5)	81 (26.2)	
RDS network size, median (IQR)^k^	25 (11–50)	20 (10–50)	30 (16–75)	<.0001^e^

Data are No. (%) except where indicated. Unknown and missing values are not displayed.

Abbreviations: HIV, human immunodeficiency virus; HPV, human papillomavirus; IQR, interquartile range; RDS, respondent-driven sampling; STI, sexually transmitted infection.

^a^Includes 57 observations with unknown vaccination status that were excluded from unvaccinated and vaccinated columns.

^b^Received zero doses or never heard of the HPV vaccine.

^c^Self-reported receipt of ≥1 dose of HPV vaccine.

^d^ *P* value comparing characteristics between vaccinated and unvaccinated individuals obtained through χ^2^ tests unless otherwise stated. Bolded *P* values indicate significance at the 5% level. *P* values exclude missing/unknown results.

^e^ *P* value obtained through Wilcoxon rank sum test.

^f^Alcohol risk classified according to the World Health Organization's Alcohol, Smoking, and Substance Involvement Screening Test as low scores, 0–10; moderate scores, 11–26; high scores, ≥27.

^g^STI diagnosis (excluding HIV): chlamydia, gonorrhea, syphilis, lymphogranuloma venereum, hepatitis A/B/C, sexually transmitted intestinal infections, herpes simplex virus 1/2, bacterial vaginosis (transmen), and anogenital warts.

^h^ *P* value obtained through Fisher exact test.

^i^Response based on the question: “In your lifetime, how many women (including transwomen) have you had sex with (vaginal, oral, or anal)?”.

^j^Response based on the question: “During the past 6 months, with how many guys have you had any kind of sex (anal, oral, mutual masturbation, rimming, frontal/vaginal, etc.)?”.

^k^Response based on the question: “How many men who have sex with men aged 16 years or older, including transmen, do you know who live or work in the [Metro Vancouver/Greater Toronto/Metro Montreal depending on site] area (whether they identify as gay or otherwise)? This includes gay/bi guys you see or speak to regularly; eg, close friends, boyfriends, spouses, regular sex partners, roommates, relatives, people you regularly hang out with, etc.”.

### HPV Vaccine Uptake

A total of 36.9% (n = 309) participants reported receiving at least 1 dose of the HPV vaccine; 63.1% received zero doses (56.3%) or had an unknown vaccination status (6.8%) (Table 1). Of those vaccinated, 41 (13.3%) reportedly received 1 dose, 69 (22.3%) 2 doses, and 170 (55.0%) 3 doses, with 29 (9.4%) reporting an unknown number of doses. The median age at first HPV vaccine dose was 23 years (IQR, 21–25 years), with 265 (85.8%) vaccinated individuals receiving their first dose before the age of 27 years. Approximately 90% of vaccinated participants reported receiving their first dose the year of or after their first oral sex encounter (Supplementary Table 2). HPV vaccination status significantly differed across the 3 cities (Montreal, 32.4% vaccinated; Toronto, 37.7% vaccinated; Vancouver, 43.5% vaccinated; *P* value .040), as did the number of doses received and age at first dose (Supplementary Table 2). Compared with those unvaccinated, more vaccinated participants had a primary health care provider, had been tested for STIs, and reported more than 10 sex partners in the past 6 months (Table 1).

### Oral HPV Prevalence

Overall, at least 1 HPV type was detected in oral samples from 22 out of the 838 participants (2.6%; 95% CI, 1.5%–3.7%; Table 2). A total of 1.2% (95% CI, .5%–1.9%) of GBM had a detectable high-risk genotype, and 1.6% (95% CI, .7%–2.4%) had a detectable low-risk genotype. Regarding vaccine-preventable types, 0.7% (95% CI, .1%–1.3%) of GBM had a detectable 4vHPV type and 1.1% (95% CI, .4%–1.8%) had a detectable 9vHPV type. Of the 4vHPV types, 0.4% of the sample had detectable HPV 16, 0.1% had HPV 18, 0.2% had HPV 6, and 0% had HPV 11 (Supplementary Table 3).

**Table 2.** Prevalence of Oral HPV Infection Among Gay, Bisexual, and Other Men Who Have Sex With Men Aged 16–30 Years, Overall and by HPV Vaccination Status, City, and Age Group, Engage, Canada, 2017 to 2019

Characteristic	N (%)	Any HPV^a^		High-Risk HPV^b^		Low- Risk HPV^c^		9vHPV^d^		4vHPV^e^		HPV 16	
		n	% (95% CI)	n	% (95% CI)	n	% (95% CI)	n	% (95% CI)	n	% (95% CI)	n	% (95% CI)
Overall	838	22	2.6 (1.5–3.7)	10	1.2 (.5–1.9)	13	1.6 (.7–2.4)	9	1.1 (.4–1.8)	6	0.7 (.1–1.3)	3	0.4 (.0–.8)
City													
Montreal	389 (46.4)	11	2.8 (1.2–4.5)	5	1.3 (.2–2.4)	7	1.8 (.5–3.1)	4	1.0 (.0–2.0)	4	1.0 (.0–2.0)	1	0.3 (.0–.8)
Toronto	212 (25.3)	3	1.4 (.0–3.0)	1	0.5 (.0–1.4)	2	0.9 (.0–2.3)	1	0.5 (.0–1.4)	0	0.0 (.0–.01)	0	0.0 (.0–.01)
Vancouver	237 (28.3)	8	3.4 (1.1–5.7)	4	1.7 (.0–3.3)	4	1.7 (.0–3.3)	4	1.7 (.0–3.3)	2	0.8 (.0–2.0)	2	0.8 (.0–2.0)
Age group, y													
16–26	459 (54.8)	11	2.4 (1.0–3.8)	6	1.3 (.3–2.4)	5	1.1 (.1–2.0)	5	1.1 (.1–2.0)	3	0.6 (.0–1.4)	1	0.2 (.0–.6)
27–30	379 (45.2)	11	2.9 (1.2–4.6)	4	1.1 (.0–2.1)	8	2.1 (.7–3.6)	4	1.1 (.0–2.1)	3	0.8 (.0–1.7)	2	0.5 (.0–1.3)
Vaccination status													
Vaccinated^f^	309 (36.9)	7	2.3 (.6–3.9)	1	0.3 (.0–1.0)	6	1.9 (.4–3.5)	2	0.6 (.0–1.5)	1	0.3 (.0–1.0)	0	0.0 (.0–.01)
Unvaccinated^g^	472 (56.3)	15	3.2 (1.6–4.8)	9	1.9 (.7–3.1)	7	1.5 (.4–2.6)	7	1.5 (.4–2.6)	5	1.1 (.1–2.0)	3	0.6 (.0–1.4)

Abbreviations: CI, confidence interval; HPV, human papillomavirus; N, overall count; n, subgroup count.

^a^Any of the following 36 types: 6/11/16/18/26/31/33/34/35/39/40/42/44/45/51/52/53/54/56/58/59/61/62/66/67/68/69/70/71/72/73/81/82/83/84/89.

^b^HPV 16/18/31/33/35/39/45/51/52/56/58/59/68.

^c^HPV 6/11/26/34/40/42/44/53/54/61/62/66/67/69/70/71/72/73/81/82/83/84/89.

^d^HPV 6/11/16/18/31/33/45/52/58.

^e^HPV 6/11/16/18.

^f^Self-reported receipt of ≥1 dose of HPV vaccine before study enrolment.

^g^Received zero doses or never heard of the HPV vaccine.

Although not statistically significantly different, point estimates of oral HPV prevalence varied by city and age (Table 2). Estimates were lower among participants in Toronto compared with Montreal and Vancouver. With respect to age, the general pattern was that oral HPV prevalence estimates were higher in GBM aged 27–30 years in comparison with those 16–26 years of age. There was a higher mean self-reported GBM network size among those with an oral HPV infection (61.4 GBM) compared with those without (41.7 GBM).

A total of 22 unique participants had detectable oral HPV, and 4 of these participants were infected with 2 HPV types each. HPV 16, HPV 44, and HPV 51 had the highest detection rates (11.5%; 3/26), followed by HPV 84 and HPV 6 (7.7%; 2/26).

### Oral HPV Prevalence by Vaccination Status

Although confidence intervals overlap and comparisons were imprecise given the rarity of these infections, compared with unvaccinated participants, those who reported at least 1 dose had lower point prevalence proportions for oral HPV (Table 2). This was true for infection with any HPV type, any high-risk HPV type, any 4vHPV type, any 9vHPV type, and for HPV 16 (Table 2). Oral HPV was not detected among the 57 participants with unknown vaccination status. Unadjusted odds ratios comparing vaccinated with unvaccinated participants were 0.3 (95% CI, .0–2.6) for oral 4vHPV infections and 0.4 (95% CI, .1–2.1) for oral 9vHPV infections. The corresponding VE estimates were 70% (95% CI, −161% to 96%) and 57% (95% CI, −110% to 91%) against 4vHPV and 9vHPV oral infections, respectively.

## DISCUSSION

We detected oral HPV infection among 2.6% (95% CI, 1.5%–3.7%) of GBM aged 16 to 30 years living in Toronto, Montreal, and Vancouver, Canada between February 2017 and August 2019. The prevalence of any high-risk type was 1.2% (95% CI, .5%–1.9%) and any 4vHPV type was 0.7% (95% CI, .1%–1.3%). Similar to previous findings [35–37], HPV 16 was one of the most commonly detected types despite our numbers being small. This is of particular importance because, among HPV-positive oral cancers (mostly oropharyngeal), HPV 16 is responsible for more than 90% of cases [38]. As anticipated, oral HPV prevalence was markedly lower than the 73% prevalence we detected for anal HPV infection, with any 4vHPV anal type infection being 27% lower in GBM who were vaccinated compared to those who were not [31].

It is notable that our estimates of oral HPV prevalence in 2017 to 2019 were considerably lower than those reported by others in previous years. In fact, among GBM aged 18 to 26 years in the United States from 2012 to 2014, oral infection with any HPV type was 8.4% among unvaccinated men without HIV [39, 40]. In another study between 2012 and 2015, oral prevalence with any HPV type was 6% in young unvaccinated GBM with HIV in the United States (mean age, 23 years) [41]. Other international studies between the years 2010 and 2012 have correspondingly reported oral HPV prevalence estimates for GMB without HIV of 8.8% for high-risk types in the Netherlands [37], and, for any HPV type, 7.0% in Australia [42] and 13.7% in the United Kingdom [43]. Higher prevalence in other studies may in part be due to differences between study populations, such as inclusion of men aged older than 30 years [37, 42], given that older age is a recognized risk factor for HPV infection [16]; variation in recruitment strategies used (including clinic settings) [39, 40, 42, 43]; differing sampling/laboratory techniques [37, 41, 42]; lower/no vaccination [39, 40, 41]; higher HIV prevalence [41]; and earlier recruitment periods [37, 39–43].

More recently, the Vaccine Impact in Men study, which surveyed GBM and transgender women aged 18 to 26 years living in Seattle, Chicago, and Los Angeles from 2016 to 2018, reported estimates nearly 3 times higher than what we observed in Engage, with oral prevalence ranging from 1.9% for any 4vHPV type, to 7.5% for any HPV type [6]. This study had similar characteristics to ours, including 40% self-reported HPV vaccination initiation and comparable specimen collection/laboratory methods. However, participants were recruited using convenience venue-based sampling from community centers and STI clinics/testing sites, which may have resulted in a cohort at higher risk for STIs [44], including HPV, than the present Engage sample that was recruited using a community sample and RDS.

Despite overlapping confidence intervals due to small numbers and unadjusted estimates, we observed lower point prevalence of oral HPV among men who self-reported at least 1 HPV vaccine dose compared with men who did not, resulting in a corresponding VE of 70% (95% CI, −161% to 96%) against prevalent 4vHPV types. This observation aligns with previously published findings in other populations. In a repeated cross-sectional study among young GBM in Melbourne, Australia, oral infections with 4vHPV were observed among 4% (7/200) of participants in the pre–gender-neutral vaccination era, compared to only 1% (1/199) in the postvaccination cohort (adjusted prevalence ratio [aPR], 0.10; 95% CI, .01–.97) [7]. Similarly, in a study conducted by Chaturvedi et al using the National Health and Nutrition Examination Survey, 4vHPV infection was 0.1% in vaccinated and 1.6% in unvaccinated participants, resulting in an adjusted VE of 88% (95% CI, 6%–98%) in the young adult US general population from 2011 to 2014 [8]. For the Vaccine Impact in Men study, Meites et al found a VE against 4vHPV vaccine-preventable oral infections of 43% (aPR, 0.57; 95% CI, −.56 to 1.70), with higher estimates for transgender women and GBM who received their first dose before or at the age of 18 (aPR, 0.13; 95% CI, −.06 to .31) [6]. Indeed, a recent systematic review observed a VE (or as the authors defined it, a “relative prevention percentage”) of 83% (95% CI, 82%–84%) for HPV vaccines on vaccine-type oral and oropharyngeal HPV infection [9].

Although less frequent among the vaccinated participants, we detected vaccine-preventable oral HPV infections among vaccinated men. Of the 2 vaccinated men with a detectable 9vHPV type, both reported that their age at first oral sex was younger than their age at their first vaccine dose, suggesting the possibility that infections were acquired prior to vaccination. These findings and others [6, 13, 32], point to the importance of getting vaccinated prior to or as soon as possible after sexual debut.

Strengths of this analysis include a large sample size, the high validity of oral specimen samples (approximately 99%), and the use of a community-recruited RDS, which allows for recruitment of diverse subgroups from populations for which random sampling would be infeasible [34]. Nevertheless, our analysis had limitations. With small (single digit) numerator counts for the oral HPV outcomes, we were unable to apply RDS weights with precision, to conduct adjusted regression analyses for VE estimation, to obtain precise VE estimates, or to examine differences by self-reported number of vaccine doses. Other limitations include the potential for misclassification of HPV vaccination status given self-report; we have previously shown, however, that misclassification would need to be extreme to bias comparisons by HPV vaccination status in the case of anal HPV infection [45]. Our findings may not be generalizable to GBM communities in smaller cities and rural areas, older GBM, or to those unwilling to participate in a sexual health study. Given the low prevalence of HIV among young men in Engage, we were unable to stratify prevalence estimates by HIV status, a risk factor for HPV infection [42]. Due to the cross-sectional nature of our analysis, time of HPV vaccination relative to infection is unknown; associations between oral infection and vaccination are therefore biased towards the null.

In conclusion, these findings, which were estimated shortly after the introduction of publicly funded HPV vaccination programs for this population, may serve as a benchmark for future studies assessing the epidemiology of oral HPV among GBM. Continued monitoring of oral HPV epidemiology will be of particular importance as vaccination coverage in GBM across Canada continues to increase [46, 47]. Given the absence of screening guidelines for oropharyngeal cancer [48], this research may further promote efforts to expand HPV vaccination coverage, especially before sexual debut, a critical step in preventing HPV-associated cancers, including oral cancer.

## Supplementary Data

Supplementary materials are available at *The Journal of Infectious Diseases* online (http://jid.oxfordjournals.org/). Supplementary materials consist of data provided by the author that are published to benefit the reader. The posted materials are not copyedited. The contents of all supplementary data are the sole responsibility of the authors. Questions or messages regarding errors should be addressed to the author.

## Supplementary Material

jiae345_Supplementary_Data

## Notes

***Acknowledgments.*** We gratefully acknowledge other members of the Engage-HPV Study Team Jody Jollimore, Rosane Nisenbaum, and Gina Ogilvie. The Engage Study (https://www.engage-men.ca/) is led by principal investigators in Toronto: Daniel Grace and Trevor Hart; Montreal: Joseph Cox and Gilles Lambert; and Vancouver: Jody Jollimore, Nathan Lachowsky, and David Moore. The Engage-HPV substudy is led by principal investigators Ann Burchell, Troy Grennan, and Alexandra de Pokomandy. HPV genotyping is supervised by Francois Coutlée. The authors thank the Engage study participants, clinical and research staff, and Community Engagement Committee members, as well as their community partner agencies. They particularly thank Julie Guenoun at le centre hospitalier de l‘Université de Montréal for conducting the human papillomavirus laboratory testing, Shayna Sparling at Toronto Metropolitan University (previously Ryerson University) for national site coordination, and Anna Yeung and Ashley Mah at the ESTIMATE Laboratory, Unity Health Toronto for research management and coordination of the Engage-HPV substudy. We also acknowledge the Applied Health Research Centre at Unity Health Toronto for data management using REDCap electronic data capture tools.

***Author contributions.*** J. A. performed formal analysis and wrote the original draft. A. N. B. contributed conceptualization and funding acquisition. A. N. B. and F. C. contributed supervision. A. N. B., F. C., J. C., D. M., R. G., C. C., and T. A. H. contributed methodology. All authors performed writing, reviewing, and editing.

***Data availability.*** Engage-HPV data are not publicly available. Data may be made available to other researchers upon request.

***Financial support.*** This work (in the Engage study) was supported by the Canadian Institutes of Health Research (CIHR; grant numbers TE2-138299, FDN-143342, and PJT-153139); the CIHR Canadian HIV Trials Network (grant number CTN300); the Canadian Association for HIV Research (grant number Engage); the Ontario HIV Treatment Network (grant number 1051); the Public Health Agency of Canada (grant number 4500345082); and an internal research grant from Toronto Metropolitan University (previously Ryerson University). The Engage-HPV substudy was funded by the CIHR (grant numbers 151944 and 148432 foundation grant to A. N. B.). The HIV/AIDS network of Fonds de Recherche du Québec-Santé supported quality assurance and control of human papillomavirus testing. R. G. was supported by a Canadian Immunization Research Network trainee scholarship. A. N. B. is a Canada Research Chair in Sexually Transmitted Infection Prevention and a recipient of a Department of Family and Community Medicine Non-Clinician Research Scientist Award, University of Toronto. N. J. L. is supported by scholar awards from the Michael Smith Foundation for Health Research (grant number 16863). T. A. H. is supported by a Chair in Gay and Bisexual Men's Health from the Ontario HIV Treatment Network. D. G. is a Tier 2 Canada Research Chair in Sexual and Gender Minority Health. D. H. S. T. is supported by a Tier 2 Canada Research Chair in HIV Prevention and STIs. T. G. is supported by a Health Professional Investigator Award from Michael Smith Health Research BC.
