# Supplementary material for: Prevalence of Oral Human Papillomavirus Infection Among Urban Gay, Bisexual, and Other Men Who Have Sex With Men in Canada, 2017–2019
Source: J Infect Dis. 2024 Jul 23;230(5):e1039–48. doi: 10.1093/infdis/jiae345 (PMC11566233; doi:10.1093/infdis/jiae345)
Supplement: jiae345_Supplementary_Data [file jiae345_supplementary_data.docx]

**Supplementary Material**

Alessandrini et al. Prevalence of Oral Human Papillomavirus Infection Among Urban Gay, Bisexual, and Other Men Who Have Sex With Men in Canada, 2017-2019


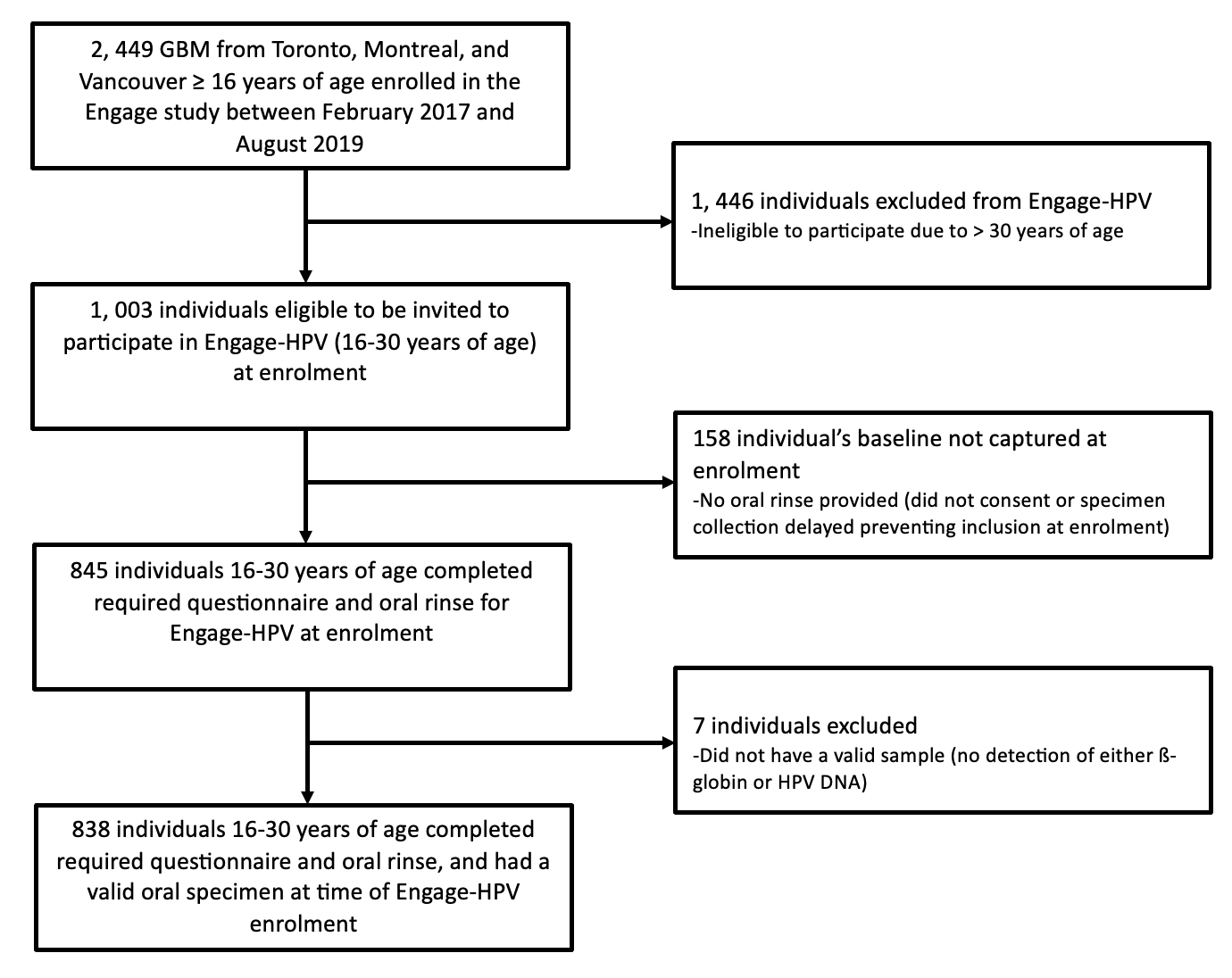


**Supplementary Figure 1:** Flow diagram illustrating inclusions and exclusions for analysis of oral HPV infection, Engage-HPV Study

**Supplementary Table 1:** Characteristics of gay, bisexual, and other men who have sex with men aged 16-30 years at enrolment, overall and according to whether or not an oral specimen was provided, Engage, Canada, 2017 to 2019

|  |  | | **Provided an Oral Specimen for HPV Testing** | | |
| --- | --- | --- | --- | --- | --- |
| **CHARACTERISTIC** | **Overall (n=1003)** | | **No (n=158)** | **Yes (n=845)** | **P** ^a^ |
| **Age, n (%)** | 550 (54.8) | 85 (53.8) | | 465 (55.0) | 0.775  0.368‡ |
| 16-26 years |  |  |  |  |  |
| 27-30 years  Median (IQR) | 453 (45.2)  26 (24-28) | 73 (46.2)  26 (24-28) | | 380 (45.0)  26 (23-28) |  |
| **City, n (%)** | 435 (43.4) | 42 (26.6) | | 393 (46.5) | **<.0001** |
| Montreal |  |  |  |  |  |
| Toronto | 249 (24.8) | 35 (22.2) | | 214 (25.3) |  |
| Vancouver | 319 (31.8) | 81 (51.3) | | 238 (28.2) |  |
| **Highest Level of Education Received, n (%)** | 192 (19.1) | 36 (22.8) | | 156 (18.5) | 0.447 |
| High school or less |  |  |  |  |  |
| Post-secondary | 646 (64.4) | 97 (61.4) | | 549 (65.0) |  |
| Graduate or professional degree | 165 (16.5) | 25 (15.8) | | 140 (16.6) |  |
| **Annual Pre-tax Income, All Sources, n (%)** | 427 (42.6) | 64 (40.5) | | 363 (43.0) | 0.784 |
| <$20,000 |  |  |  |  |  |
| $20,000-$39,999 | 314 (31.3) | 47 (29.7) | | 267 (31.6) |  |
| $40,000-$59,999 | 174 (17.3) | 31 (19.6) | | 143 (16.9) |  |
| $60,000-$79,999 | 66 (6.6) | 11 (7.0) | | 55 (6.5) |  |
| $80,000+ | 22 (2.2) | 5 (3.2) | | 17 (2.0) |  |
| **Ethnicity, n (%)** |  |  | |  | **0.002** |
| Indigenous | 13 (1.3) | 0 (0.0) | | 13 (1.5) |  |
| Black, African, Caribbean | 29 (2.9) | 7 (4.4) | | 22 (2.6) |  |
| Asian | 125 (12.5) | 34 (21.5) | | 91 (10.8) |  |
| English or French Canadian | 458 (45.7) | 61 (38.6) | | 397 (47.0) |  |
| Other European | 182 (18.1) | 27 (17.1) | | 155 (18.3) |  |
| Other or mixed | 196 (19.5) | 29 (18.4) | | 167 (19.8) |  |
| **Sexual Orientation, n (%)** | 784 (78.2)  219 (21.8) | 120 (76.0)  38 (24.0) | | 664 (78.6)  181 (21.4) | 0.463 |
| Gay  Bisexual, queer, straight, questioning, asexual, pansexual, two-spirit |  |  |  |  |  |
|  |  |  | |  |  |
| **Gender Identity, n (%)** | 918 (91.5) | 146 (92.4) | | 772 (91.4) | 0.861 |
| Cis-gender man |  |  |  |  |  |
| Transgender man | 18 (1.8) | 3 (1.9) | | 15 (1.8) |  |
| Gender queer, gender non-conforming, two-spirit | 67 (6.7) | 9 (5.7) | | 58 (6.9) |  |
| **Has a Primary Healthcare Provider, n (%)** |  |  | |  | 0.815 |
| Yes | 601 (59.9) | 96 (60.8) | | 505 (59.8) |  |
| No | 402 (40.1) | 62 (39.2) | | 340 (40.2) |  |
| **Current Smoking Status, n (%)** | 297 (29.6) | 40 (25.3) | | 257 (30.4) | 0.148 |
| Never smoker |  |  |  |  |  |
| Current smoker | 464 (46.3) | 71 (44.9) | | 393 (46.5) |  |
| Former smoker | 235 (23.4) | 46 (29.1) | | 189 (22.4) |  |
| Unknown | 7 (0.7) | 1 (0.6) | | 6 (0.7) |  |
| **Alcohol Risk (ASSIST Score)** ^b^**, n (%)** | 618 (61.6) | 91 (57.6) | | 527 (62.4) | 0.376 |
| Lower risk |  |  |  |  |  |
| Moderate risk | 291 (29.0) | 49 (31.0) | | 242 (28.6) |  |
| High risk | 57 (5.7) | 12 (7.6) | | 45 (5.3) |  |
| Unknown | 37 (3.7) | 6 (3.8) | | 31 (3.7) |  |
| **Cannabis Use, Lifetime, n (%)** | 146 (14.6) | 25 (15.8) | | 121 (14.3) | 0.630 |
| No |  |  |  |  |  |
| Yes | 849 (84.6) | 132 (83.5) | | 717 (84.8) |  |
| Unknown | 8 (0.8) | 1 (0.6) | | 7 (0.8) |  |
| **Poppers Use, Lifetime, n (%)** | 440 (43.9) | 67 (42.4) | | 373 (44.1) | 0.700 |
| No |  |  |  |  |  |
| Yes | 552 (55.0) | 89 (56.3) | | 463 (54.8) |  |
| Unknown | 11 (1.1) | 2 (1.3) | | 9 (1.1) |  |
| **Tested for STIs, Lifetime, n (%)** | 98 (9.8) | 17 (10.8) | | 81 (9.6) | 0.657 |
| No |  |  |  |  |  |
| Yes | 896 (89.3) | 140 (88.6) | | 756 (89.5) |  |
| Unknown | 9 (0.9) | 1 (0.6) | | 8 (0.9) |  |
| **Self-Reported STI Diagnosis** ^c^**, Lifetime, n (%)** | 441 (44.0) | 71 (44.9) | | 370 (43.8) | 0.704 |
| No |  |  |  |  |  |
| Yes | 539 (53.7) | 82 (51.9) | | 457 (54.1) |  |
| Unknown | 23 (2.3) | 5 (3.2) | | 18 (2.1) |  |
| **Laboratory-confirmed HIV Infection, n (%)** | 944 (94.1) | 148 (93.7) | | 796 (94.2) | 0.492 |
| Negative |  |  |  |  |  |
| Positive  Missing/ Inconclusive | 53 (5.3)  6 (0.6) | 8 (5.1)  2 (1.3) | | 45 (5.3)  4 (0.5) |  |
| **Ever Had Oral Sex With a Man (Giving or Receiving), Lifetime, n (%)** | 2 (0.2) | 1 (0.6) | | 1 (0.1) | 0.290† |
| No |  |  |  |  |  |
| Yes | 1001 (99.8) | 157 (99.4) | | 844 (99.9) |  |
| **Age at First Oral Sex With a Man (Giving or Receiving), median (IQR)**  Missing | 17 (15-19)  3 | 16 (14-18)  1 | | 17 (15-19)  2 | 0.087‡ |
| **Ever Had Sex With a Woman, Lifetime** ^d^**, n (%)** | 651 (64.9) | 102 (64.6) | | 549 (65.0) | 0.920 |
| No |  |  |  |  |  |
| Yes | 352 (35.1) | 56 (35.4) | | 296 (35.0) |  |
| **Number of Male Sex Partners** ^e^**, Past 6 Months, n (%)** | 127 (12.7) | 22 (13.9) | | 105 (12.4) | 0.948 |
| 0-1 partners |  |  |  |  |  |
| 2-5 partners | 358 (35.7) | 54 (34.2) | | 304 (36.0) |  |
| 6-10 partners | 227 (22.6) | 36 (22.8) | | 191 (22.6) |  |
| >10 partners | 291 (29.0) | 46 (29.1) | | 245 (29.0) |  |
| **Number of Male Oral/Anal Sex Partners, Past 6 Months, n (%)** | 144 (14.4) | 25 (15.8) | | 119 (14.1) | 0.950 |
| 0-1 partners |  |  |  |  |  |
| 2-5 partners | 373 (37.2) | 57 (36.1) | | 316 (37.4) |  |
| 6-10 partners | 222 (22.1) | 35 (22.2) | | 187 (22.1) |  |
| >10 partners | 264 (26.3) | 41 (25.9) | | 223 (26.4) |  |
| **Current Regular Partner, n (%)** |  |  | |  | 0.340 |
| Yes | 473 (47.2) | 80 (50.6) | | 393 (46.5) |  |
| No | 530 (52.8) | 78 (49.4 | | 452 (53.5) |  |
| **Oral Sex (Given to a Man), Past 6 Months,**  **n (%)** |  |  | |  | 0.121 |
| Yes | 967 (96.4) | 149 (94.3) | | 818 (96.8) |  |
| No | 36 (3.6) | 9 (5.7) | | 27 (3.2) |  |
| **Rimming (Given to a Man), Past 6 Months,**  **n (%)** |  |  | |  | 0.365 |
| Yes | 667 (66.5) | 110 (69.6) | | 557 (65.9) |  |
| No | 336 (33.5) | 48 (30.4) | | 288 (34.1) |  |
| **Number of HPV Doses, Lifetime,**  **n (%)** |  |  | |  | 0.556 |
| Unvaccinated/Unknown vaccination status | 639 (63.7) | 106 (67.1) | | 533 (63.1) |  |
| 1 Dose | 49 (4.9) | 7 (4.4) | | 42 (5.0) |  |
| 2 Doses | 77 (7.7) | 8 (5.1) | | 69 (8.2) |  |
| 3 Doses | 205 (20.4) | 33 (20.9) | | 172 (20.4) |  |
| Vaccinated, unknown number of doses | 33 (3.3) | 4 (2.5) | | 29 (3.4) |  |
| **Age Group at First HPV Vaccine Dose,**  **n (%)** |  |  | |  | 0.625 |
| Unvaccinated/ Unknown vaccination status  ≤26 Years | 639 (63.7)  311 (31.0) | 106 (67.1)  44 (27.8) | | 533 (63.1)  267 (31.6) |  |
| 27-30 Years | 37 (3.7) | 6 (3.8) | | 31 (3.7) |  |
| Vaccinated, unknown age at first dose | 16 (1.6) | 2 (1.3) | | 14 (1.7) |  |
| Median (IQR) | 23 (21-25) | 23 (21-25) | | 23 (21-25) | 0.444‡ |
| **Vaccinated Before Age at First Oral Sex With a Man (Giving or Receiving), n (%)** |  |  | |  | 0.585 |
| Unvaccinated/ Unknown vaccination status | 639 (63.7) | 106 (67.1) | | 533 (63.1) |  |
| Yes  No  Vaccinated, missing vaccination or oral sex age | 11 (1.1)  337 (33.6)  16 (1.6) | 1 (0.6)  49 (31.0)  2 (1.3) | | 10 (1.2)  288 (34.1)  14 (1.7) |  |
| **RDS Network Size** ^f^**, median (IQR)** | 25 (11-50) | 30 (15-50) | | 25 (11-50) | 0.300‡ |

*IQR* interquartile range, *HPV* human papillomavirus, *HIV* human immunodeficiency virus, *STI* sexually transmitted infection, *RDS* respondent-driven sampling.

‘Unknown’ reflects don’t know/ can’t remember unless otherwise stated.

† P-value obtained through Fisher’s exact test.

‡ P-value obtained through Wilcoxon Rank Sum Test.

^a^ P-value comparing characteristics between included and excluded individuals obtained through chi-square tests unless otherwise stated. Bolded p-values indicate significance at the 5% level. P-values exclude missing/unknown results.

^b^ Alcohol risk classified according to the World Health Organization’s Alcohol, Smoking, and Substance Involvement Screening Test as Low scores: 0-10; moderate scores; 11-26, high scores; ≥27.

^c^ STI diagnosis (excluding HIV): chlamydia, gonorrhea, syphilis, LGV, hepatitis A/B/C, sexually transmitted intestinal infections, herpes simplex virus 1/2, bacterial vaginosis (trans men), anogenital warts.

^d^ Response based on the question: “In your lifetime, how many women (including trans women) have you had sex with (vaginal, oral or anal)?”.

^e^ Response based on the question: “During the PAST 6 MONTHS, with how many guys have you had any kind of sex (anal, oral, mutual masturbation, rimming, frontal/vaginal, etc.)?”

^f^ Response based on the question: “How many men who have sex with men aged 16 years or older, including trans men, do you know who live or work in the [Metro Vancouver/Greater Toronto/Metro Montreal depending on site] area (whether they identify as gay or otherwise)? This includes gay/bi guys you see or speak to regularly; e.g., close friends, boyfriends, spouses, regular sex partners, roommates, relatives, people you regularly hang out with, etc.”.

**Supplementary Table 2**: Characteristics of gay, bisexual, and other men who have sex with men aged 16-30 years at enrolment, overall and by city, Engage, Canada, 2017 to 2019

| **CHARACTERISTIC** | | **Overall**  **(n= 838)** | **Montreal (n= 389)** | **Toronto (n= 212)** | **Vancouver (n= 237)** | **P** ^a^ |
| --- | --- | --- | --- | --- | --- | --- |
| **RDS Network Size** ^b^**, median (IQR)** | 25 (11-50) | | 25 (11-50) | 30 (15-60) | 25 (10-50) | 0.234‡ |
| **Age, n (%)** | | |  |  |  | 0.113 |
| 16-26 Years | | 459 (54.8) | 228 (58.6) | 108 (50.9) | 123 (51.9) |  |
| 27-30 Years  Median (IQR) | | 379 (45.2)  26 (23-28) | 161 (41.4)  26 (23-28) | 104 (49.1)  26 (24-28) | 114 (48.1)  26 (24-28) | 0.186‡ |
| **Self-Reported HPV Vaccination Status** ^c^**, n (%)** | |  |  |  |  | **0.040** |
| Vaccinated (≥1 dose) | | 309 (36.9) | 126 (32.4) | 80 (37.7) | 103 (43.5) |  |
| Unvaccinated | | 472 (56.3) | 230 (59.1) | 121 (57.1) | 121 (51.0) |  |
| Unknown | | 57 (6.8) | 33 (8.5) | 11 (5.2) | 13 (5.5) |  |
| **Number of Doses, Lifetime, n (%)** | | |  |  |  | **0.026** |
| Unvaccinated/ Unknown vaccination status | | 529 (63.1) | 263 (67.6) | 132 (62.3) | 134 (56.5) |  |
| 1 Dose | | 41 (4.9) | 15 (3.8) | 13 (6.1) | 13 (5.5) |  |
| 2 Doses | | 69 (8.2) | 38 (9.8) | 13 (6.1) | 18 (7.6) |  |
| 3 Doses | | 170 (20.3) | 61 (15.7) | 51 (24.1) | 58 (24.5) |  |
| Vaccinated, unknown number of doses | | 29 (3.5) | 12 (3.1) | 3 (1.4) | 14 (5.9) |  |
| **Age Group at First HPV Vaccine Dose, n (%)** | |  |  |  |  | **0.011** |
| Unvaccinated/ Unknown vaccination status | | 529 (63.1) | 263 (67.6) | 132 (62.3) | 134 (56.5) |  |
| ≤26 years | | 265 (31.6) | 116 (29.8) | 62 (29.2) | 87 (36.7) |  |
| 27-30 years | | 30 (3.6) | 7 (1.8) | 13 (6.1) | 10 (4.2) |  |
| Vaccinated, unknown age at first dose | | 14 (1.7) | 3 (0.8) | 5 (2.4) | 6 (2.5) |  |
| Median (IQR) | | 23 (21-25) | 23 (21-25) | 24 (22-26) | 24 (21-25) | **0.034**‡ |
| **Vaccinated Before Age at First Oral Sex With a Man (Giving or Receiving), n (%)** | |  |  |  |  | 0.086† |
| Unvaccinated/ Unknown vaccination status | | 529 (63.1) | 263 (67.6) | 132 (62.3) | 134 (56.5) |  |
| Yes | | 10 (1.2) | 3 (0.8) | 2 (0.9) | 5 (2.1) |  |
| No | | 285 (34.0) | 120 (30.8) | 73 (34.4) | 92 (38.8) |  |
| Vaccinated, missing vaccination or oral sex age | | 14 (1.7) | 3 (0.8) | 5 (2.4) | 6 (2.5) |  |

*IQR* interquartile range, *HPV* human papillomavirus, *RDS* respondent-driven sampling.

† P-value obtained through Fisher’s exact test.

‡ P-value obtained through Kruskal-Wallis test.

^a^ P-value comparing characteristics between recruitment cities obtained through chi-square tests unless otherwise stated. Bolded p-values indicate significance at the 5% level. P-values exclude missing/unknown results.

^b^ Response based on the question: “How many men who have sex with men aged 16 years or older, including trans men, do you know who live or work in the [Metro Vancouver/Greater Toronto/Metro Montreal depending on site] area (whether they identify as gay or otherwise)? This includes gay/bi guys you see or speak to regularly; e.g., close friends, boyfriends, spouses, regular sex partners, roommates, relatives, people you regularly hang out with, etc.”.

^c^ Self-reported receipt of ≥1 dose of HPV vaccine= vaccinated and received zero doses or never heard of the HPV vaccine= unvaccinated.

**Supplementary Table 3**: Genotype-specific oral HPV prevalence among gay, bisexual, and other men who have sex with men aged 16 to 30 Years, overall and by HPV vaccination status, Engage, Canada, 2017 to 2019

| **HPV Type** | **Overall** ^a^  **(n= 838)** | **Vaccinated** ^b^  **(n= 309)** | **Unvaccinated** ^c^  **(n= 472)** |
| --- | --- | --- | --- |
|  | **n (%)** | **n (%)** | **n (%)** |
| HPV6 | 2 (0.2) | 1 (0.3) | 1 (0.2) |
| HPV11 | 0 (0.0) | 0 (0.0) | 0 (0.0) |
| HPV16 | 3 (0.4) | 0 (0.0) | 3 (0.6) |
| HPV18 | 1 (0.1) | 0 (0.0) | 1 (0.2) |
| HPV26 | 1 (0.1) | 1 (0.3) | 0 (0.0) |
| HPV31 | 0 (0.0) | 0 (0.0) | 0 (0.0) |
| HPV33 | 1 (0.1) | 0 (0.0) | 1 (0.2) |
| HPV34 | 0 (0.0) | 0 (0.0) | 0 (0.0) |
| HPV35 | 0 (0.0) | 0 (0.0) | 0 (0.0) |
| HPV39 | 0 (0.0) | 0 (0.0) | 0 (0.0) |
| HPV40 | 1 (0.1) | 0 (0.0) | 1 (0.2) |
| HPV42 | 1 (0.1) | 0 (0.0) | 1 (0.2) |
| HPV44 | 3 (0.4) | 1 (0.3) | 2 (0.4) |
| HPV45 | 1 (0.1) | 0 (0.0) | 1 (0.2) |
| HPV51 | 3 (0.4) | 0 (0.0) | 3 (0.6) |
| HPV52 | 0 (0.0) | 0 (0.0) | 0 (0.0) |
| HPV53 | 0 (0.0) | 0 (0.0) | 0 (0.0) |
| HPV54 | 0 (0.0) | 0 (0.0) | 0 (0.0) |
| HPV56 | 0 (0.0) | 0 (0.0) | 0 (0.0) |
| HPV58 | 1 (0.1) | 1 (0.3) | 0 (0.0) |
| HPV59 | 0 (0.0) | 0 (0.0) | 0 (0.0) |
| HPV61 | 1 (0.1) | 0 (0.0) | 1 (0.2) |
| HPV62 | 0 (0.0) | 0 (0.0) | 0 (0.0) |
| HPV66 | 0 (0.0) | 0 (0.0) | 0 (0.0) |
| HPV67 | 0 (0.0) | 0 (0.0) | 0 (0.0) |
| HPV68 | 1 (0.1) | 0 (0.0) | 1 (0.2) |
| HPV69 | 0 (0.0) | 0 (0.0) | 0 (0.0) |
| HPV70 | 0 (0.0) | 0 (0.0) | 0 (0.0) |
| HPV71 | 0 (0.0) | 0 (0.0) | 0 (0.0) |
| HPV72 | 1 (0.1) | 1 (0.3) | 0 (0.0) |
| HPV73 | 1 (0.1) | 0 (0.0) | 1 (0.2) |
| HPV81 | 0 (0.0) | 0 (0.0) | 0 (0.0) |
| HPV82 | 1 (0.1) | 1 (0.3) | 0 (0.0) |
| HPV83 | 1 (0.1) | 1 (0.3) | 0 (0.0) |
| HPV84 | 2 (0.2) | 1 (0.3) | 1 (0.2) |
| HPV89 | 0 (0.0) | 0 (0.0) | 0 (0.0) |

*HPV* human papillomavirus.

^a^ Observations with unknown vaccination status included in overall column.

^b^ Self-reported receipt of ≥1 dose of HPV vaccine.

^c^ Received zero doses or never heard of the HPV vaccine.
